# Wnt Signaling Pathway Linked to Intestinal Regeneration via Evolutionary Patterns and Gene Expression in the Sea Cucumber *Apostichopus japonicus*

**DOI:** 10.3389/fgene.2019.00112

**Published:** 2019-02-19

**Authors:** Jianbo Yuan, Yi Gao, Lina Sun, Songjun Jin, Xiaojun Zhang, Chengzhang Liu, Fuhua Li, Jianhai Xiang

**Affiliations:** ^1^CAS Key Laboratory of Experimental Marine Biology and CAS Key Laboratory of Marine Ecology and Environmental Sciences, Institute of Oceanology, Chinese Academy of Sciences, Qingdao, China; ^2^Laboratory for Marine Biology and Biotechnology and Marine Ecology and Environmental Science, Qingdao National Laboratory for Marine Science and Technology, Qingdao, China; ^3^Center for Ocean Mega-Science, Chinese Academy of Sciences, Qingdao, China

**Keywords:** sea cucumber, intestinal regeneration, Wnt signaling pathway, positive selection, natural selection

## Abstract

Many echinoderms are regenerative species that exhibit exceptional regenerative capacity, and sea cucumber is a representative organism that could regenerate the whole intestine after evisceration. There are many signaling pathways participate in the regeneration process, but it is not clear which is essential for the intestinal regeneration. In this study, we performed genome-wide comprehensive analyses on these regeneration-related signaling pathways, and found the Wnt signaling pathway was one of the most conservative pathways among regenerative species. Additionally, among these signaling pathways, we found that the Wnt signaling pathway was the only one under positive selection in regenerative echinoderms, and the only one enriched by differentially expressed genes during the intestinal regeneration. Thus, it suggests both coding sequence and gene expression of the Wnt signaling pathway have been shaped by natural selection to provide the genetic architecture for intestinal regeneration. *Wnt7, Fz7*, and *Dvl* are the three positively selected genes and also happen to be three upstream genes in the Wnt signaling pathway. They are all significantly upregulated at the early stages of regeneration, which may contribute significantly to the early activation of Wnt signaling and the initiation of intestinal regeneration. Expression knockdown of *Wnt7* and *Dvl* by RNA interference significantly inhibit intestinal extension, implying that they are essential for intestinal regeneration. As an important regeneration-related gene, the downstream gene *c-Myc* is also conserved and highly expressed during the whole regeneration stages, which may make the Wnt/c-Myc signaling to be an important way to promote intestinal regeneration. Therefore, it is reasonable to conclude that the Wnt signaling pathway is the chosen one to play an important role in intestinal regeneration of sea cucumbers, or even in the regeneration of other echinoderms.

## Introduction

In 2005, *Science* has running a series of articles on 125 scientific issues, and organ regeneration is one of the top 25 topics that still deserving global attention today. Organ regeneration has been investigated in various organisms, but the capacity is varied among different phyla and even highly divergent between closely related species (Brockes and Kumar, 2008). Unlike the non-regenerative animals, regenerative species displayed strong regenerative potentials that can regenerate missed organs or body parts (Agata and Inoue, 2012). Among the widely investigated regenerative animals, echinoderm is one of the major groups of deuterostomes that can quickly renew most injured organs (Garcia-Arraras et al., 1998; Bannister et al., 2005; Candelaria et al., 2006). However, even belong to the same phyla, different echinoderms displayed significantly different regenerative capacities. Brittle stars, starfishes, sea cucumbers, and crinoids were considered as regenerative species, whereas sea urchins have relative poor regenerative capacity that can only regenerate amputated tube feet and spines (Heatfield and Travis, 1975; Garcia-Arraras et al., 1998; Patruno et al., 2003; Candelaria et al., 2006; Agata and Inoue, 2012). It is well-known that the arms of starfishes can be regenerated after amputation (Rubilar et al., 2005; Shibata et al., 2010). And sea cucumber is considered to be an excellent model for studying organ regeneration, which possesses a striking capacity to regenerate the whole intestine after evisceration (Conant, 1973; Candelaria et al., 2006; Dolmatov and Ginanova, 2009).

There are many signaling pathways participate in organ regeneration, including pathways of JAK-STAT, MAPK, TGF-β, Wnt, PI3K-Akt, Hippo, Hedgehog, and so on. The first five are essential signaling pathways involved in regulating pluripotency of stem cells, which will activate the self-renewal capacity of basic cells and generate all the cell types (Burdon et al., 1999; Okita and Yamanaka, 2006; Nostro et al., 2011). Among them, Wnt and TGF-β signaling pathways are two widely investigated pathways that not only integrally involved in both stem cell and cancer cell maintenance, but also participated in growth of intestinal and epidermal systems (Mishra et al., 2005; Reya and Clevers, 2005; Espada et al., 2009; Trompouki et al., 2011). The Hippo signaling pathway is highly conserved and plays a critical role in tissue homeostasis by inhibiting the transcription co-activators YAP, TAZ, and Yki (Zhao et al., 2011; Mo et al., 2014; Yu et al., 2015). It is also reported to have important functions in self-renewal of stem cells and intestinal regeneration (Cai et al., 2010; Yu et al., 2015). The Hedgehog signaling pathway is well-known for its roles in development of cancer, and also critical for the regeneration of liver, prostate, and other tissues (Karhadkar et al., 2004; Evangelista et al., 2006; Ochoa et al., 2010). There are many transcriptomics and proteomics studies suggested that some genes of these signaling pathways upregulated during intestinal regeneration of sea cucumber, implying their important roles in organ regeneration of echinoderms (Sun et al., 2011, 2017). However, due to the limited genomic resources of echinoderms previously, the functions of these signaling pathways on echinoderm organ regeneration lacks systemic studies.

Evisceration and intestinal regeneration is a defensive strategy shared by many sea cucumbers. The sea cucumber *Apostichopus japonicus* is an ideal animal for the research on organ regeneration. It can discard its intestine, and rapidly regenerate them with normal functions within a few weeks (Mashanov and Garcia-Arraras, 2011). Organ regeneration is a process that involves wound healing, cell migration, proliferation, differentiation, and organ remodeling, which may be under positive selection pressure (Balabaud et al., 2004; Delorme et al., 2012; Zhang et al., 2017). Some positively selected genes and stem cells have been identified to contribute to organ regeneration in regenerative animals (Balabaud et al., 2004; Liu et al., 2015). As there are so many signaling pathways participate in organ regeneration, it is of interest to investigate whether some of them, under selective pressure, are critical for the intestinal regeneration of sea cucumbers.

**FIGURE 1:**
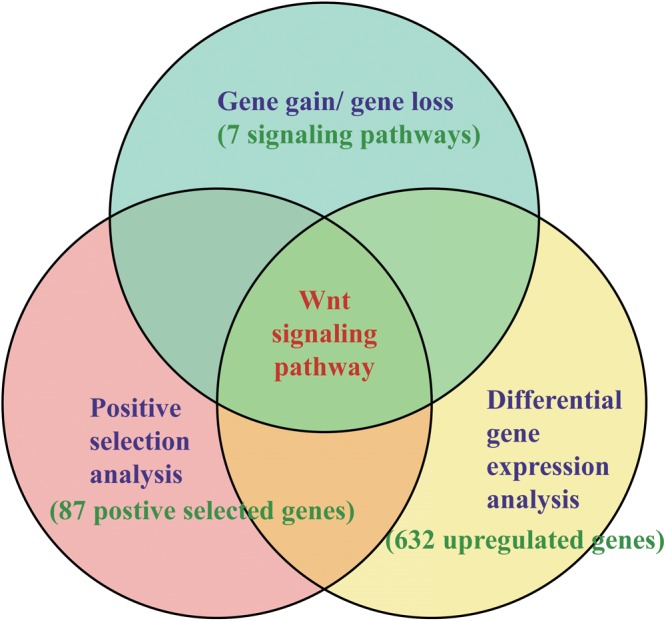
Methods of identifying key pathways and genes in regeneration. The methods combined the results of gene gain/gene loss analysis, positive selection analysis, and DGE analysis.

Although many functional genes have been identified, the molecular and genetic mechanisms of intestinal regeneration need further investigation (Mashanov et al., 2010, 2012; Sun et al., 2013). With the benefit of the recently published genome of the sea cucumber *A. japonicus* and starfish *Acanthaster planci* (Hall et al., 2017; Zhang et al., 2017), it provides valuable genomic resources for the study of organ regeneration of echinoderms. In this study, a thorough investigation has been performed on these regeneration-related signaling pathways among echinoderms. An integrative result of gene gain/loss analysis, positive selection analysis, DGE analysis, and expression knockdown experiments, suggested the positive selection on the Wnt signaling pathway may make significant contribution to the intestinal regeneration of sea cucumbers (Figure 1).

## Materials and Methods

### Genome Resources

The genome sequences and the full gene information of 13 species were collected from the genome database of NCBI^1^, including three echinoderms, *A. japonicus* (MRZV00000000.1), *A. planci* (BDGF00000000.1), *Strongylocentrotus purpuratus* (AAGJ00000000.5), a hemichordate *Saccoglossus kowalevskii* (ACQM00000000.1), three representative regenerative animals, *Danio rerio* (GCA_000002035.4), *Hydra vulgaris* (ACZU00000000.1), and *Amphimedon queenslandica* (ACUQ00000000.1), and six animals with relative poor regeneration capacity, *Drosophila melanogaster* (GCA_000001215.4), *Bombyx mori* (GCA_000151625.1), *Daphnia pulex* (GCA_000187875.1), *Branchiostoma floridae* (GCA_000003815.1), *Crassostrea gigas* (GCA_000297895.1), and *Caenorhabditis elegans* (GCA_000002985.3). The intestinal regeneration transcriptome data of *A. japonicus* were collected from the SRA database of NCBI with the accession numbers of SRR5083072, SRR5083087, SRR5083077, SRR5083088, SRR5083079, and SRR5083080 (Zhang et al., 2017). The RNA samples of the transcriptome were extracted from the regenerative intestine at 3, 7, 10, 14, and 21 days post-evisceration and a normal intestine prior to evisceration.

### Gene Gain/Loss Analysis

In order to identify the consensus signaling pathways among regenerative animals, gene gain and loss analysis was performed on the genes involved in the seven signaling pathways that participate in organ regeneration, including pathways of JAK-STAT, MAPK, TGF-β, Wnt, PI3K-Akt, Hippo, Hedgehog. Full genes of 12 species above (except *S. kowalevskii*) were collected for mapping the KEGG (Kyoto Encyclopedia of Genes and Genomes) pathways. Then, we detect the presence/absence status for each gene among different signaling pathways.

### Positive Selection Analysis

The peptide sequences of three echinoderms were clustered by the Markov clustering program orthoMCL (Li et al., 2003). All the peptide sequences were compared by an all versus all BLASTP with a threshold value of E-value ≤ 1 × 10^-05^ and subsequently clustered by MCL with an inflation value of 1.5. The single-copy orthologous gene families were clustered among three echinoderm genomes and used for the positive selection analysis.

To assess the contribution of natural selection on the single-copy genes in regenerative echinoderms, the ratios (*ω*) of non-synonymous substitution per non-synonymous site (d_N_) to synonymous substitution per synonymous site (d_S_) were calculated using the software package PAML (version 4.48a). Pair-wise maximum likelihood analysis were performed in runmode = -2. We excluded alignments that had d_S_ larger than 2 to minimize statistical artifacts from saturation effects in d_S_. The homologs were aligned using ClustalX and the conserved sites were extracted using Gblocks (Thompson et al., 2002; Talavera and Castresana, 2007). A phylogenetic tree (*A. planci* #1, *A. japonicus* #1, *S purpuratus*), was used for the positive selection analysis, which assigned regenerative echinoderms (*A. planci* and *A. japonicus*) as the foreground branch. The branch model and branch-site model were used to detect positive selection along the foreground branch (Yang, 1998; Yang and Nielsen, 1998). In the branch model tests, the null model M0 assumes the ratio *ω* is invariable, and the alterative model allows *ω* vary in different branches. LRT were applied to test the significant differences between the alterative and null model. In the branch-site model tests, the BEB was used to calculate the posterior probabilities (*p*-value) for site classes, and identify the amino acid sites under positive selection. The amino acid sites may be under positive selection if the *p*-value is ≧ 95%.

### Phylogenetic Analysis and Gene Family Comparison

The *Wnt* and *Frizzled* gene families of the three echinoderms and *S. kowalevskii* were collected according to the annotated functions from GenBank. In order to select other homologous genes of the *Wnt* and *Frizzled* gene families of the four species, a BLAST alignment was made to align the full protein-coding genes to the genes of the *Wnt* and *Frizzled* gene family with an E-value cutoff of 1E-07. Subsequently, the putative genes were validated by Pfam and SMART (Letunic et al., 2015; Finn et al., 2016). Phylogenetic analysis was conducted on these *Wnt* and *Frizzled* genes to cluster them into various subfamilies. The amino acid sequences of the *Wnt* and *Frizzled* genes were aligned using MUSCLE 3.6 (Edgar, 2004). Then, the ML methods were used for phylogenetic tree construction. The substitution models that best fit the alignment data were estimated with jModelTest 2 (Darriba et al., 2012). ML analysis was performed using PhyML with the substitution model of JTT + gamma + Inv (Guindon and Gascuel, 2003). 1000 bootstraps were conducted to produce the branch support values (Li et al., 2011; Zhu et al., 2011). Based on the alignment results above, bayesian phylogenetic inference (BI) was performed with the help of the program Mrbayes 3.2.1 (Ronquist and Huelsenbeck, 2003). In the BI analysis, two independent runs, each with four chains, were calculated for millions of generations until the standard deviation of split frequencies converged toward zero. The first 25% of sampled trees was discarded as burn-in.

### Differential Gene Expression Analysis and Gene Ontology

The transcriptome data of the entire intestinal regeneration (non-eviscerated control and 3, 5, 7, 14, 21d after evisceration) of *A. japonicus* were used for the DGE analysis (Zhang et al., 2017). Tophat2 (version 2.0.14) was used to map the sequencing reads to the genome (Trapnell et al., 2009), and Cufflinks (version 2.2.1) was used to calculate expected FPKM as expression values for each gene (Trapnell et al., 2012).

For positively selected genes and DEGs, we conducted KEGG pathway enrichment analysis using KAAS [87,88]. Functional enrichment analysis was performed using Omicshare Cloud Tools^2^, enriched KEGG Orthology (KO) terms were calculated relative to the background of full protein-coding genes.

### Real-Time PCR Analysis

In order to validate the DGE results and detect gene expression level at the early stages (1d after evisceration), we performed quantitative real-time PCR on four genes (*Wnt7, Wnt8, Fz7*, and *Dvl*). NADH dehydrogenase was set as the internal control gene. Total RNA was extracted from the regenerated intestinal tissues at 1, 3, and 14 d after evisceration, and from the intestinal tissues of non-eviscerated sea cucumbers that served as controls. Then, isolated RNA samples were treated by DNase using the RNeasy Mini Kit and RNase-Free DNase Set (Qiagen), and qualified by agarose gel electrophoresis and quantified using a Nanodrop spectrophotometer (Thermo Fisher Scientific). RNA samples were reversely transcribed into cDNA using random hexamers and Superscript II reverse transcriptase for first-strand cDNA synthesis. The synthesized cDNAs were diluted with RNase-free water and stored at -80°C.

The primers of the four genes were designed using Primer3 (v0.4.0^3^) (Supplementary Table S1). The relative expression levels of the four genes were determined using the SYBR Green real-time PCR assay on an Eppendorf Mastercycler ep realplex thermocycler (Eppendorf, Hamburg, Germany). Thermal cycling was as follows: 95°C for 5 s, 40 cycles at 95°C for 10 s, 60°C for 20 s, and 72°C for 30 s (Sun et al., 2017). In comparison to NADH dehydrogenase (internal control), the 2^-ΔΔCT^ method was used to calculate the expression level. Statistical analysis was performed using SPSS 18 software (SPSS, Inc., Chicago, IL, United States).

### RNA Interference (RNAi) and Inhibitor Experiments

To elucidate the functions of the Wnt signaling pathway in the intestinal regeneration of sea cucumbers, we conducted RNAi experiments on *Wnt7* and *Fz7*, and compared with that of a specific inhibitor (salinomycin) of the Wnt signaling pathway. Healthy adults of *A. japonicus* were collected from the coast of Qingdao, Shandong Province with an average body wet weight of 16.10 ± 3.80 g (mean ± SE), and acclimated in tanks for 1 week at the temperate of 16–18°C. Three pairs of dsRNA primers were designed on *Wnt7, Dvl*, and *EGFP* (negative control), and the PCR products (*Wnt7* of 527 bp and *Dvl* of 430 bp) were generated with a T7 promoter anchored to the two primers (Supplementary Table S2). The PCR products of *EGFP* (289 bp) were amplified from the pEGFP-N1 plasmid. The PCR products were purified using Gel Extraction Kit (OMEGA, Japan). dsRNA was synthesized with TranscriptAid T7 High Yield Transcription Kit (Thermo Fisher Scientific, United States), according to the manufacturer’s protocol.

We optimized the effective silencing dose of each dsRNA by injecting three different dosages, 4, 8, and 16 μg. In order to improve the interference efficiency, dsRNA was mixed with the *in vivo* transfection reagent (EntransterTM-*in vivo*; Engreen, Beijing, China) with the ratio of 2:1 (dsRNA: transfection reagent). The mixed solutions were diluted to 100 μl of PBS solution, and injected into the coelom of *A. japonicus*. The intestine from each treatment was collected for RNA extraction after 48 h. Expression levels of each gene were determined by real-time PCR, and the optimal silencing doses were identified according to the differential expression level of each gene.

Sea cucumbers were divided into three groups: ds*Wnt7* group (*n* = 6), ds*Dvl* group (*n* = 6), and ds*EGFP* group (*n* = 6). All groups were induced evisceration by injecting 1.5 ml KCl (0.35 M) into the coelom. The optimal dosage of 16 μg of dsRNA was used for the preparation of the mixed solution. The first time of the solutions injection was at 6 h post-evisceration. Then, the injection was repeated for four times in every 2 days. After 7 days, we recorded the length of the body and intestine of each individual. The average body length was 5.37 ± 0.72 cm. To minimize the effects of individual differences, we formatted the intestine length with the formula of L_int_/L_body_ = F_int_/5.37, which formatted the body length of 5.37 cm. L_int_ and L_body_ indicated the observed length of intestine and body, respectively, and F_int_ indicated the formatted intestine length.

Salinomycin is a specific inhibitor of the Wnt signaling cascade, with the target site of the Wnt/Fz/LRP complex (Lu et al., 2011). For the expression knockdown experiments of the inhibitor, sea cucumbers were divided into three groups: the groups injected with 10 mg/kg (salinomycin/body weight) and 2 mg/kg of salinomycin, and the control group (100 μl PBS with 2% DMSO). Salinomycin (MedChemExpress, HY-15597) was pre-dissolved in 100 μl PBS (with 2% DMSO). The injection was also repeated for four times in every 2 days. The independent samples *t*-test was used to indicate the significant bias between different groups with the significance level of *p* < 0.05.

## Results

### Conserved Signaling Pathways Among Regenerative Animals

**FIGURE 2:**
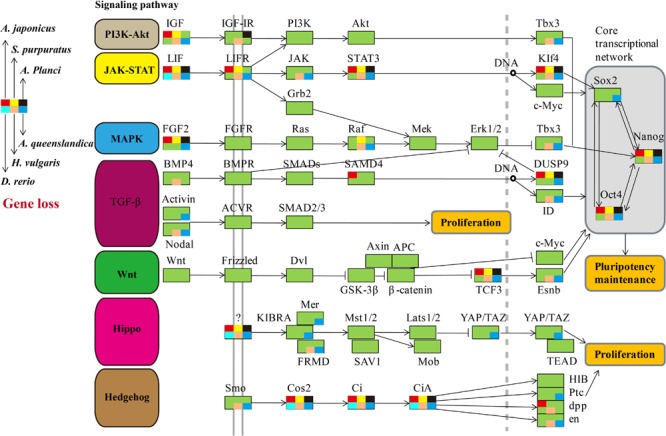
Comparative analysis of genes in regeneration-related signaling pathways of regenerative animals. The comparative analysis are performed on seven regeneration-related signaling pathways of six species. The genes present in genomes are markered in green background, and the genes absent in genomes are markered in other colors according to the legends.

We comprehensively searched for the genes involved in the seven regeneration-related signaling pathways of the three echinoderms and other three representative regenerative animals (Figure 2). Genes of the six species were universally distributed among the seven signaling pathways except for the JAK-STAT and Hedgehog signaling pathway, which with most of genes have not been identified, including *LIF, LIFR, STAT3, KIf4, Cos2, Ci*, and *CiA*. As for the other five signaling pathways, some of genes have not been detected among regenerative species, such as *IGF, Tbx3, FGF2, Raf, DUSP9, Nodal, TCF3, Esnb, FRMD, Smo, en*, and so on. However, the Wnt signaling pathway, including genes of *Wnt, Frizzled, Dvl, GSK-3β, β-catenin, Axin, APC*, and *c-Myc*, is the only pathway contains genes that all detected among regenerative species. It was also conserved in the genome of sea urchin *S. purpuratus*, a species with weak regeneration capacity. However, the Wnt signaling pathway was less conservative (eg., *c-Myc*) among many non-regenerative species (Supplementary Figure S1). TCF3 and Esnb in the downstream of the Wnt signaling pathway was another way to perform its function relative to c-Myc, however, some of them have not been identified in regenerative species. Thus, the pathway of Wnt/c-Myc signaling is one of the most conserved signaling pathways among regenerative animals.

### Positive Selection on the Wnt Signaling Pathway

Comparative genomic analyses were conducted among three echinoderm species, and 31,976 families of homologous genes were clustered. The mean rates of d_S_ and d_N_ among three echinoderms were around 1.50 and 0.07, respectively (Supplementary Figure S2). There were 2,807 conserved single-copy orthologous gene families used for positive selection analysis. Based on the branch model test, we identified 87 positively selected genes (LRT *p*-value ≦ 0.05) in the foreground branches (regenerative echinoderms *A. planci* and *A. japonicus*). Functional enrichment analysis indicated these positively selected genes were majorly enriched in protein export (ko03060) and Wnt signaling pathway (ko04310) (Supplementary Table S3). Except for Wnt signaling pathway, there was rare or none of positive selected genes identified in the other six regeneration-related signaling pathways. *Wnt7, Fz7*, and *Dvl* were the three positively selected genes identified in the Wnt signaling pathway (Table 1), which also just the three upstream genes that initiate the signal transduction (Figure 2). And accidently, Fz7 is the receptor of Wnt7 (Von Maltzahn et al., 2011). These three genes were under strong positive selection with ω1 > 1, especially for *Dvl*, which with ω1 = 3.502 (LRT, *p* = 0.009), and eight sites (21P, 31H, 34V, 40Q, 69Y, 96S, 125Q, and 133I) were identified under positive selection in branch-site model test. Therefore, the Wnt signaling pathways may be positively selected for the regeneration process in regenerative echinoderms.

**Table 1:** Positive selected genes related to Wnt signaling pathway and protein export.

Genes	Branch models					Branch-site models			
	B0:lnL	B0: ω0 (ω1 = 1)	BA: lnL	BA: ω0, ω1^∗^	*p*-Value	p0	p2a	ω2	Positively selected sites (BEB)
*Wnt7*	–2001.701	0.036	–1999.715	0.035, 1.164	0.046	0.495	0.505	1.838	
*Fz7*	–3294.404	0.086	–3292.234	0.0589, 300.088	0.037	0.330	0.000	1.000	
*Dvl*	–1760.390	1.000	–1757.014	0.135, 3.502	0.009	0.024	0.976	9.404	21P 31H 34V 40Q 69Y 96S 125Q 133I
*OXA1L*	–2604.090	0.048	–2607.372	0.0736, 1	0.010	0.000	0.627	1.000	64D 74T 78I 138N 175S 213M
*SRP68*	–2197.410	0.612	–2194.960	0.083, 1	0.027	0.066	0.934	999.000	
*Tmem59*	–2362.254	0.077	–2358.819	0.158, 11.936	0.009	0.271	0.000	1.000	
*Tmem243*	–693.650	1.000	–691.282	0.254, 999	0.030	0.032	0.208	1.000	
*SLC25A38*	–1236.724	1.000	–1233.414	1, 999	0.010	0.014	0.144	999.000	5A 19M 44L 46P 52T 85R 98V
*SLC25A39*	–1675.127	0.099	–1666.093	0.086, 119.818	0.000	0.617	0.383	28.501	

*^∗^ω: the ratios of non-synonymous substitution per non-synonymous site to synonymous substitution per synonymous site; ω0, ω1: background and foreground ω values, respectively. The amino acid sites under positive selection were identified using BEB.*

Protein supply and transportation are one of the essential processes in regeneration, especially for wound healing and new tissue formation. Many proteins related to substance transport and metabolism have been identified to be upregulated at the translational level during intestinal regeneration (Sun et al., 2017). Except for the two positively selected genes (*OXA1L* and *SRP68*) take part in protein export (ko03060), there were several genes (*Tmem59, Tmem243, SlC25A38*, and *SLC25A39*) related to protein transportation identified to be under positive selection (Table 1). *Tmem59* and *Tmem243* were two genes both encoding transmembrane protein. Both *SlC25A38* and *SLC25A39* encode solute carrier family 25, which is a glycine transporter that imports glycine into the mitochondrial matrix. The enrichment of positively selected genes in protein transportation indicates the important roles of protein supply in regeneration.

### Identification and Phylogenetic Classification of *Wnt* and *Frizzled* Gene Family

**FIGURE 3:**
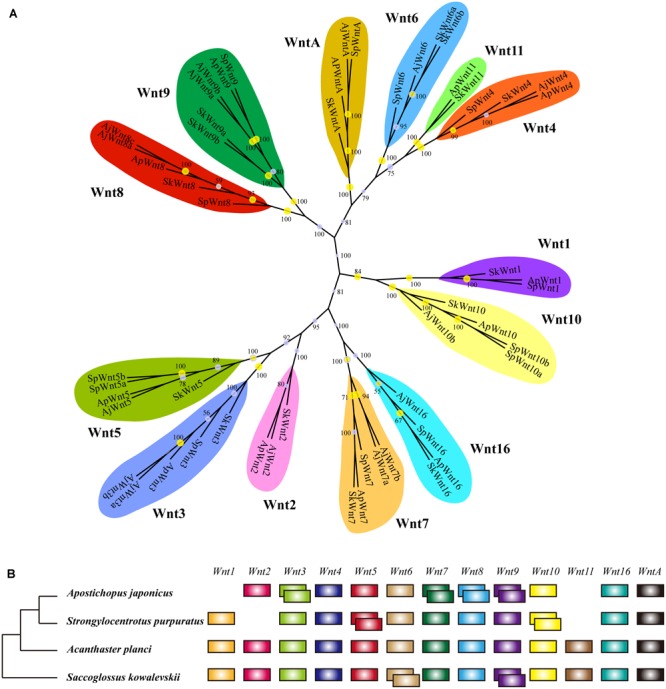
Wnt gene family in echinoderms. **(A)** Phylogenetic tree of the Wnt gene family of three echinoderms and *Saccoglossus kowalevskii*. The phylogenetic tree is constructed using the maximum likelihood (ML) method with 1000 bootstraps and Bayesian inference (BI). Yellow circles indicate the support values of ML analysis larger than 80%. The support values of BI analysis are displayed beside each node. **(B)** Comparison of Wnt gene family members across echinoderms and *S. kowalevskii*. Empty indicate the loss of particular Wnt genes, and overlapping boxes represent duplicated Wnt genes.

As the conservation and positive selection both identified in the Wnt signaling pathway, we analyzed the composition of the gene families on this pathway. Among the three upstream genes, *Dvl* was a single-copy gene among echinoderms, while *Wnt* and *Frizzled* were large gene families with many members involved. After homologous search and phylogenetic classification, *Wnt* genes were further classified into 13 subfamilies (Figure 3A), which was consistent with previous results (Robert et al., 2014). The 13 *Wnt* genes were almost present in echinoderms except for several genes, such as *Wnt1* and *Wnt11* in *A. japonicus*, and *Wnt2* and *Wnt11* in *S. purpuratus* (Figure 3B). *Wnt11* also has not been identified in other sea urchins (Robert et al., 2014), indicating it may be lost in the ancestor of Echinozoa. The 13 *Wnt* genes were all single-copy in *A. planci*, but several genes were duplicated in *A. japonicus*, including the positively selected gene *Wnt7*.

Phylogenetic analyses have classified *Frizzled* genes into eight clusters (*Fz1*-*Fz5, Fz7, Fz8, Fz10*) (Supplementary Figure S3). It was different from previous researches, which classified *Frizzled* genes into five subfamilies (*Fz1/2/7, Fz3/6, Fz4, Fz5/8*, and *Fz9/10*) according to their complexity and diversity (Robert et al., 2014). Besides, unlike their results (*Fz3* lost in sea urchins), we identified a *Fz3* (XP_011667363) in *S. purpuratus* that clustered with other *Fz3* of echinoderms in a monophyletic branch. The distribution of *Frizzled* genes was similar among three echinoderms. *Fz3/6* and *Fz9/10* were single copy, and *Fz1/2/7, Fz4*, and *Fz5/8* were multi-copy. In comparison to *A. planci, Fz1/2/7* was expanded in two echinozoans, *A. japonicus* and *S. purpuratus*. When comparing the *Wnt* and *Frizzled* gene families between *S. purpuratus* and regenerative echinoderms (*A. planci* and *A. japonicus*), no obvious gene expansion has been identified in regenerative echinoderms, which indicates the gene dosage was not positively selected with respect to regeneration.

### Enrichment of Differentially Expressed Genes (DEGs) During Intestinal Regeneration

In order to evaluate the effects of gene expression on echinoderm regeneration, we conducted DGE analyses on the transcriptomes of the intestinal regeneration of *A. japonicus*. A total of 632 DEGs were identified upregulated during the intestinal regeneration (Fold change > 4, *p* ≦ 0.01, and *q* ≦ 0.05). Functional enrichment analysis indicated these DEGs were majorly enriched in glutathione metabolism (ko00480), ribosome (ko03010), biosynthesis of unsaturated fatty acid (ko01040), cytochrome P450 (ko00982, ko00980), and so on (Supplementary Figure S4). Among the seven regeneration-related signaling pathways, the Wnt signaling pathway was the only one with DEGs enriched (seven DEGs, RichFactor = 0.05, *p* ≦ 0.05).

**FIGURE 4:**
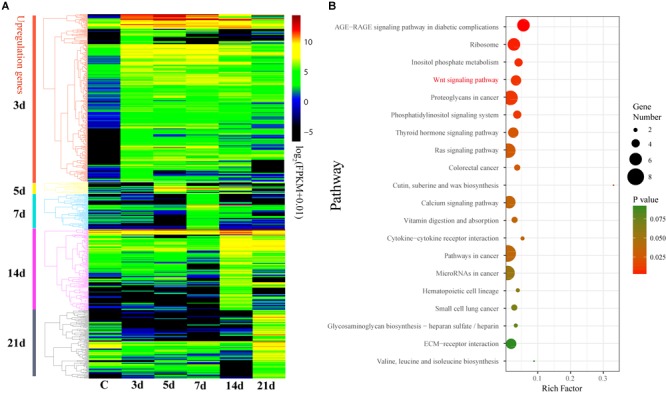
Differential expression analysis of genes during intestinal regeneration. **(A)** Expression profiles of the DEGs at different intestinal regeneration time points: 0 (C), 3 day (3d), 5 day (5d), 7 day (7d), 14 day (14d), 21 day (21d). DEGs are divided into five groups that significantly expressed at 3d (3d versus C), 5d (5d versus 3d), 7d (7d versus 5d), 14d (14d versus 7d), and 21d (21d versus 14d). **(B)** Significantly enriched pathways of DEGs that significantly upregulated at 3d. RichFactor is the ratio of the number of DEGs in this pathway term to the number of all genes in this pathway term.

These 632 DEGs displayed different expression patterns that beyond our expectations. Unlike the expression pattern of the DEGs that upregulated at 3d (3d versus C), the DEGs that upregulated at 5, 7, 14, and 21d were mostly specifically highly expressed during those stages (Figure 4A and Supplementary Figure S5), indicating their specific functions during the intestinal regeneration. However, the DEGs that upregulated at 3d were highly expressed during the whole regeneration stages (5–14d), and the number of DEGs (286 genes) was significantly more than those upregulated at other stages (22∼119 genes). It implies these genes were functionally active all the time and may be more important for the intestinal regeneration of *A. japonicus*. Besides, these 286 DEGs that upregulated at 3d have different enriched functions in comparison to all the 632 DEGs (Figure 4B). They were enriched in KEGG terms of ribosome (ko03010), inositol phosphate metabolism (ko00562), signaling pathways of Wnt (ko04310), phosphatidy linositol (ko04070), thyroid hormone (ko04919), Ras (ko04014), calcium (ko04020), and some pathways related to cancer (ko05205, ko05210, ko05200, ko05206, ko05222), which showed a strong correlation with regeneration processes, such as wound healing, cell migration, proliferation, differentiation, and organ remodeling. As expected, among the seven regeneration-related signaling pathways, the Wnt signaling pathway was still the only one with DEGs enriched among these 286 DEGs, and it was one of the top five KO terms that showed significantly enrichment (*p* ≦ 0.01). Thus, the Wnt signaling pathway played prominent roles in intestinal regeneration at the transcriptional level.

### Expression Patterns of Genes Involving the Wnt Signaling Pathway

**FIGURE 5:**
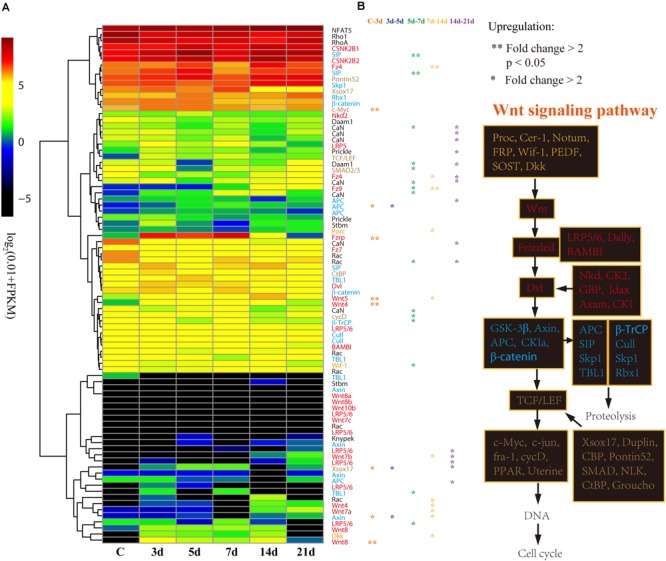
Differential expression analysis of Wnt signaling pathway genes. **(A)** Expression profiles of the DEGs at different intestinal regeneration time points: 0 (C), 3 day (3d), 5 day (5d), 7 day (7d), 14 day (14d), 21 day (21d). Genes with different colors are correspondent to the color of genes in the plot B. **(B)** Genes involved in Wnt signaling pathway. Genes from upstream to downstream of the Wnt signaling pathway are in different color.

With respect to its importance, we analyzed the expression patterns of genes involved in the Wnt signaling pathway in detail. According to the expression profiles, the 85 genes of the Wnt signaling pathway were clustered into four groups from low to high expression level (Figure 5A). Generally, the upstream genes displayed relatively low expression level, such as *Wnt* and *LRP5/6*, and the downstream genes showed relatively high expression level, such as *c-Myc* and *cycD* (Figure 5B). However, the DEGs that significantly upregulated at 3d (Fold change > 2, *p* < 0.05) were primarily upstream genes, including *Wnt4, Wnt5, Wnt8*, and *Fzrp*. As a key gene in the Wnt signaling pathway, *c-Myc* was also significantly upregulated at 3d (Figure 5A). Besides, *Wnt, TCF/LEF, Fzrp, Frizzled, Dvl, Dkk*, and *c-Myc* were the key genes in the Wnt signaling pathway, and they were all upregulated at 3d, and highly expressed during the whole regeneration stages (Supplementary Figure S6).

**FIGURE 6:**
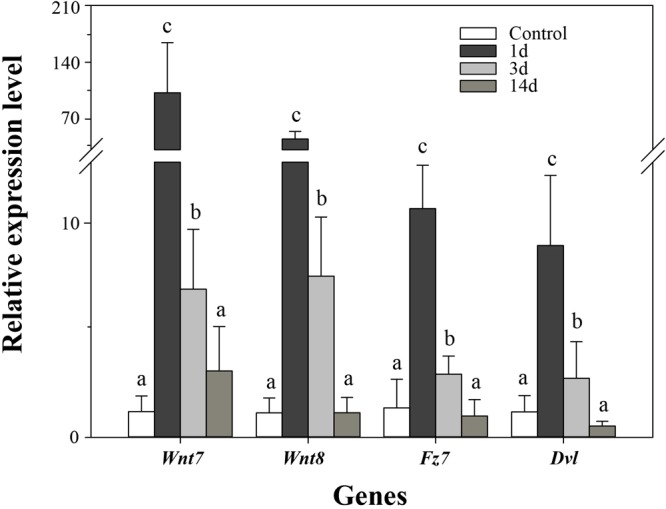
Real-time PCR analysis of four genes during intestinal regeneration. Normal tissues of the intestine were treated as the control group. The values were normalized against NADH. Data are the mean ± standard deviation of the triplicate experiments. Different lowercase letters indicate significant differences (*p* < 0.05).

In order to validate the results of DGE analysis, we analyzed mRNA expression of four genes using real-time PCR. Consistent with the DGE results, the four genes were upregulated at 3d after evisceration, but some of them downregulated at 14d (Figure 6). In addition, the DGE results were also consistent with previous real-time PCR results of some *Wnt* genes (Ortiz-Pineda et al., 2009; Sun et al., 2013; Li et al., 2017). As inferred from DGE results, the key genes of the Wnt signaling pathway were upregulated at the early stages of intestinal regeneration, possibly even earlier than 3d. Besides, two of the three positively selected genes in the Wnt signaling pathway, *Wnt7* and *Fz7*, have not been identified significantly upregulated in the DGE analysis. Thus, we performed real-time PCR on these genes to examine the expression at the early stage (1d) of regeneration. As expected, the expression of the three positively selected genes, *Wnt7, Fz7*, and *Dvl*, were all significantly upregulated at 1d after evisceration (*p* < 0.05), which even exceed the expression level at 3d. Therefore, the Wnt signaling pathway may perform its function at the initial stages of the intestinal regeneration.

### Functional Approval of *Wnt7* and *Dvl*

**FIGURE 7:**
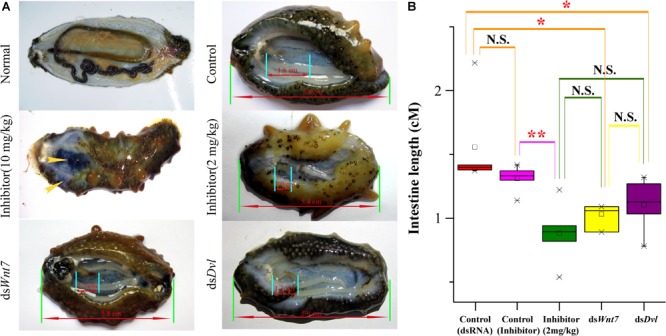
The intestinal regeneration after injection of dsRNA and inhibitor. **(A)** Photos of the intestinal regeneration after injection of inhibitor (10 and 2 mg/kg), ds*Wnt7*, ds*Dvl*, and ds*EGFP* (Control). As the performance of the negative controls of dsRNA and inhibitor were similar, only the control of dsRNA was displayed. **(B)** Distributions of the intestinal lengths of different treatment groups. Independent samples *t*-test was used to test the significant differences. ^∗∗^*p* < 0.01, ^∗^*p* < 0.05, and N.S., significant differences.

To further elucidate the essential functions of the Wnt signaling pathway, we performed RNAi experiments on these positively selected genes after evisceration. The silencing efficiency test indicated that the expression level of *Wnt7* and *Dvl* was significantly downregulated under the injection of the optimal dosage (16 μg) of dsRNA (Supplementary Figure S7). The expression knockdown of *Wnt7* and *Dvl* significantly inhibited the intestinal regrowth in comparison to that injection of ds*EGFP*, suggesting *Wnt7* and *Dvl* were functional important in intestinal regeneration (Figure 7).

As a specific inhibitor of the Wnt signaling pathway, salinomycin displayed significantly inhibition of the intestinal regrowth (average intestine length of 0.88 cm), which was even stronger than that of ds*Wnt7* (average intestine length of 1.11 cm) and *dsDvl* (average intestine length of 1.07 cm), although no significance were noted. However, the inhibitor could not only inhibit intestinal regeneration, but also affects other physiological processes that the sea cucumbers injected by inhibitor tend to autolysis, especially for the high dose (10 mg/kg) of inhibitor. The body wall of these sea cucumbers became soft and the coelomic visceral epithelium became dull (Figure 7A). Salinomycin could not only target the Wnt7/Fz7/LRP complex, but also target other Wnt/Fz/LRP compounds, which may correspondent with other physiological processes. Unlike that of the inhibitor, the injection of ds*Wnt7* and ds*Dvl* displayed similar phenotypic with the control group except for intestine regeneration. Therefore, it was reasonable to conclude that Wnt7 was specifically essential for the intestinal regeneration of sea cucumbers.

## Discussion

In the regenerative species, there are several signaling pathways participate in their regeneration processes. Different species may have specific regeneration-related signaling pathways, or regenerate tissues via a combination of several signaling pathways. Sea cucumber is a regenerative species with strong regeneration capacity, however, which signaling pathway participates in the intestinal regeneration remains unclear. Thus, in this study, we performed genome-wide comprehensive studies on these signaling pathways, and detected the Wnt signaling pathway is the chosen one that plays critical roles in the intestinal regeneration of sea cucumber. When comparing with other regeneration-related signaling pathways, the Wnt signaling pathway was one of the most conserved pathways among regenerative species, and was the only one under positive selection and enriched by DEGs during the intestinal regeneration. Thus, these results suggested that both coding sequence and gene expression of the Wnt signaling pathway have been shaped by natural selection to provide the genetic architecture for intestinal regeneration in sea cucumbers.

The Wnt signaling pathway is a well-studied pathway that has been identified to participate in stem cell signaling network and growth in intestinal systems (Kuhnert et al., 2004; Reya and Clevers, 2005; Espada et al., 2009). *Wnt, Frizzled*, and *Dvl* are the three upstream genes in the Wnt signaling pathway, and the binding of Wnt ligands to receptors of the Frizzled family is the first step in activation of Wnt signaling (Robert et al., 2014). Some *Wnt* genes (eg., *Wnt6* and *WntA*) have been identified involved in the intestinal regeneration of sea cucumbers (Sun et al., 2013; Li et al., 2017). Other genes, including *Wnt4, Fz1/2/7*, and *Fz5/8*, were identified upregulated during 5–10 days after evisceration and might participate in regulation of tissue and organ formation during intestinal regeneration (Girich et al., 2017). However, in this study, *Wnt7, Fz7*, and *Dvl* were the only three genes that identified to be positively selected in the Wnt signaling pathway. Interestingly, Fz7 is the receptor of Wnt7, and the components could activate the planar-cell-polarity pathway and exposure dramatically stimulates on the symmetric expansion of satellite stem cells (Von Maltzahn et al., 2011). Satellite stem cells are capable of self-renewal and long-term reconstitution of the satellite cell niche in mediating the growth and regeneration of skeletal muscle (Kuang et al., 2007). Over expression of Wnt7 could lead to a marked enhancement of the regeneration process during muscle regeneration (Kuang et al., 2007). However, the functions of Wnt7 and Fz7 were majorly studied in the regeneration of muscle and hair follicles (Kuang et al., 2007; Von Maltzahn et al., 2011; Dong et al., 2017), whereas our study suggested they were participated in intestinal regeneration. Furthermore, our results indicated *Wnt7, Fz7*, and *Dvl* were significantly upregulated at the initial stages (1d or maybe earlier) of regeneration. The earlier expression of *Wnt* and *Frizzled* genes may be responsible for the earlier activation of Wnt signaling. Thus, the positive selection of the three genes may make a great contribution to the early activation of Wnt signaling, which will promote the initiation of the biological functions of the Wnt signaling pathway during the intestinal regeneration of sea cucumbers. Furthermore, RNAi experiments of *Wnt7* and *Dvl* also supported these genes were essential for the intestinal regeneration. Injection of Wnt/Fz inhibitor could not only inhibit the intestinal regrowth, but also affect the normal physiological processes of sea cucumber. Whereas, unlike the phenotypic performance of the inhibitor, RNAi of *Wnt7* and *Dvl* could only inhibit the intestinal regrowth, implying *Wnt7* may be specifically essential for the intestinal regeneration.

In the KEGG pathway of signaling pathways regulating pluripotency of stem cells (ko04550), Wnt signaling pathway performs its function through a transcription factor, Esnb, which encodes estrogen related receptor beta. However, the functions of Esnb in regeneration are still unclear at present. Esnb and its regulator TCF3 have not been identified in some regenerative species (Figure 2), and no obvious differential expression was detected for Esnb during intestinal regeneration of sea cucumber. Whereas, another transcription factor, c-Myc, has been identified to be upregulated by Wnt signaling pathway to promote the cell proliferation previously (Zhang et al., 2012). Besides, the downstream of Wnt/c-Myc signaling was identified to be required for the intestinal regeneration (Ashton et al., 2010). In consistent with their results, we also found that c-Myc was significantly upregulated at 3d after evisceration, and highly expressed during the whole regeneration stages. By modifying the expression of its target genes, c-Myc could result for numerous biological effects, such as cell proliferation, cell growth, apoptosis, differentiation, and stem cell self-renewal (Evan et al., 1992; Wilson et al., 2004; Zhang et al., 2012). Thus, c-Myc may be essential for the intestinal regeneration of sea cucumbers, and Wnt/c-Myc signaling may be the important way to promote intestinal regeneration.

## Author Contributions

JX and FL conceived and designed the study. LS and XZ collected the data. JY, YG, and CL conducted the bioinformatics analyses. LS performed RT-PCR experiments. YG and SJ performed RNAi and inhibitor experiments. JY wrote the manuscript. XZ revised the manuscript. All authors read and approved the final manuscript.

## Conflict of Interest Statement

The authors declare that the research was conducted in the absence of any commercial or financial relationships that could be construed as a potential conflict of interest.

## Footnotes

1 https://www.ncbi.nlm.nih.gov/

2 http://www.omicshare.com/tools/?l=en-us

3 http://bioinfo.ut.ee/primer3-0.4.0/primer3/

## Abbreviations

BEB: Bayes empirical Bayes approach
DEGs: differentially expressed genes
DGE: differential gene expression
d_N_: non-synonymous substitution per non-synonymous site
d_S_: synonymous substitution per synonymous site
FDR: false discovery rate
FPKM: fragments per kilobase of transcript per million mapped reads
KO: KEGG Orthology
LRT: Likelihood ratio tests
ML: maximum likelihood
SMART: Simple Modular Architecture Research Tool
