# Supplementary material for: Wnt Signaling Pathway Linked to Intestinal Regeneration via Evolutionary Patterns and Gene Expression in the Sea Cucumber Apostichopus japonicus
Source: Front Genet. 2019 Feb 19;10:112. doi: 10.3389/fgene.2019.00112 (PMC6390002; doi:10.3389/fgene.2019.00112)

Supplementary Material

Wnt signaling pathway linked to intestinal regeneration via evolutionary patterns and expression in the sea cucumber *Apostichopus japonicus*

Jianbo Yuan[1,2,3,&], Yi Gao[1,2, 3,&], Lina Sun[1,2,3], Songjun Jin[1,2,3], Xiaojun Zhang[1,2, 3,*], Chengzhang Liu[1,2,3], Fuhua Li[1,2,3] and Jianhai Xiang [1,2,3,*]

1. CAS Key Laboratory of Experimental Marine Biology & CAS Key Laboratory of Marine Ecology and Environmental Sciences, Institute of Oceanology, Chinese Academy of Sciences, Qingdao 266071, China

2. Laboratory for Marine Biology and Biotechnology & Marine Ecology and Environmental Science, Qingdao National Laboratory for Marine Science and Technology, Qingdao 266071, China

3. Center for Ocean Mega-Science, Chinese Academy of Sciences, Qingdao, 266071, China

*** Correspondence:**
Prof. Jianhai Xiang

Institute of Oceanology, Chinese Academy of Sciences.

7, Nanhai Road, Qingdao 266071, China.

Tel: + 86-532-82898568. Fax: + 86-532-82898578.

E-mail: fhli@qdio.ac.cn

Dr. Xiaojun Zhang

Institute of Oceanology, Chinese Academy of Sciences.

7, Nanhai Road, Qingdao 266071, China.

E-mail: xjzhang@qdio.ac.cn

Supplementary Table S1. The primer sequences used in the real-time PCR.

Gene ID	Primer sequence
qPCR-NADH-F	GTCCTACGACCCAATCTGGA
qPCR-NADH-R	ATGAGCCTTGGTTACGTTGG
qPCR-Wnt7-F	GGTTCCTGCACCACACAA
qPCR-Wnt7-R	AATGATTTTCCGAGCGTTC
qPCR-Wnt8-F	GCTGCGTCGTAGAATGTCAA
qPCR-Wnt8-R	ATCTCTCGGGAGGTTCTGGT
qPCR-Dvl-F	GGTTACTTCACCATTCCT
qPCR-Dvl-R	ATTCTTTTTCTGATTCCG
qPCR-Fz7-F	CAGGACGACGCTGGACTT
qPCR-Fz7-R	TGGAAATGCTTCACAGGCTA

Supplementary Table S2. The primer sequences used in the dsRNA silencing.

Gene ID	Primer sequence
dsWnt7-T7F	TAATACGACTCACTATAGGCCGAAGTAAGGATACCGCC
dsWnt7-T7R	TAATACGACTCACTATAGGAACCCCTACCACAACACATAAGT
dsDvl-T7F	TAATACGACTCACTATAGGCATCAACCAGGTCCAATC
dsDvl-T7R	TAATACGACTCACTATAGGATTTACGAGCATCCCTTC
dsFz7-T7F	TAATACGACTCACTATAGGACAGGACGACGCTGGACT
dsFz7-T7R	TAATACGACTCACTATAGGCATCCCACCCAGTATCG
dsEGFP-F	TAATACGACTCACTATAGGGCAGTGCTTCAGCCGCTACCC
dsEGFP-R	TAATACGACTCACTATAGGGAGTTCACCTTGATGCCGTTCTT

Supplementary Table S3. KEGG enrichment of positively selected genes.

Pathway ID	Pathway	DEGs genes (19)	All genes(2506)	P value
ko03060	Protein export	2 (10.53%)	19 (0.76%)	0.009
ko04310	Wnt signaling pathway	3 (15.79%)	84 (3.35%)	0.024
ko03070	Bacterial secretion system	1 (5.26%)	4 (0.16%)	0.030
ko03010	Ribosome	3 (15.79%)	98 (3.91%)	0.036
ko05034	Alcoholism	2 (10.53%)	42 (1.68%)	0.039
ko05200	Pathways in cancer	4 (21.05%)	182 (7.26%)	0.044

Supplementary Fig. S1. Comparative analysis of genes in regeneration-related signaling pathways of animals with poor regeneration capacity. The comparative analysis are performed on seven regeneration-related signaling pathways of six species. The genes present in genomes are markered in green background, and the genes absent in genomes are markered in other colors according to the legends.

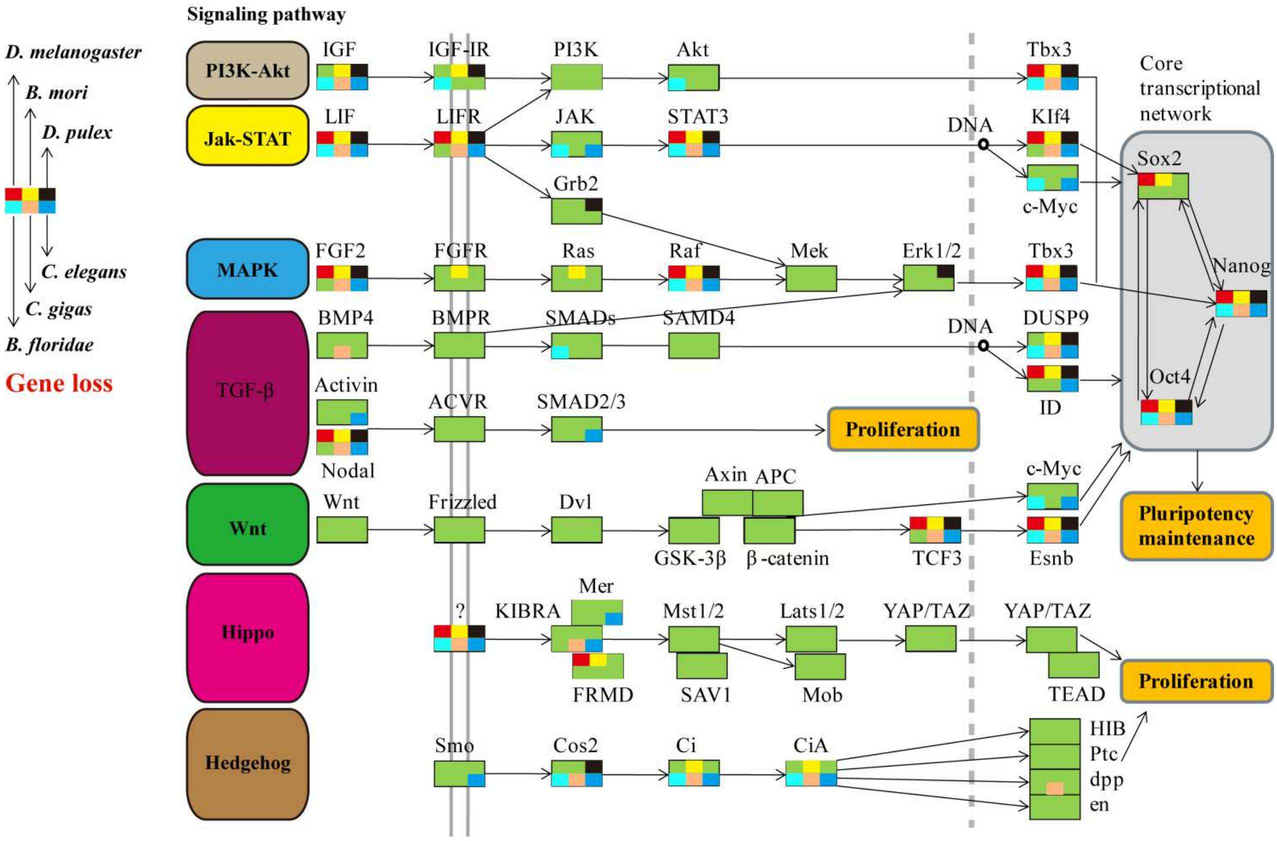

Supplementary Fig. S2. The distribution patterns of synonymous (d_S) and non-synonymous substitution rate (d_N) in comparisons among three echinoderms: *A. japonicus* (Ajap), *S. purpuratus* (Spur), and *A. planci* (Apla).

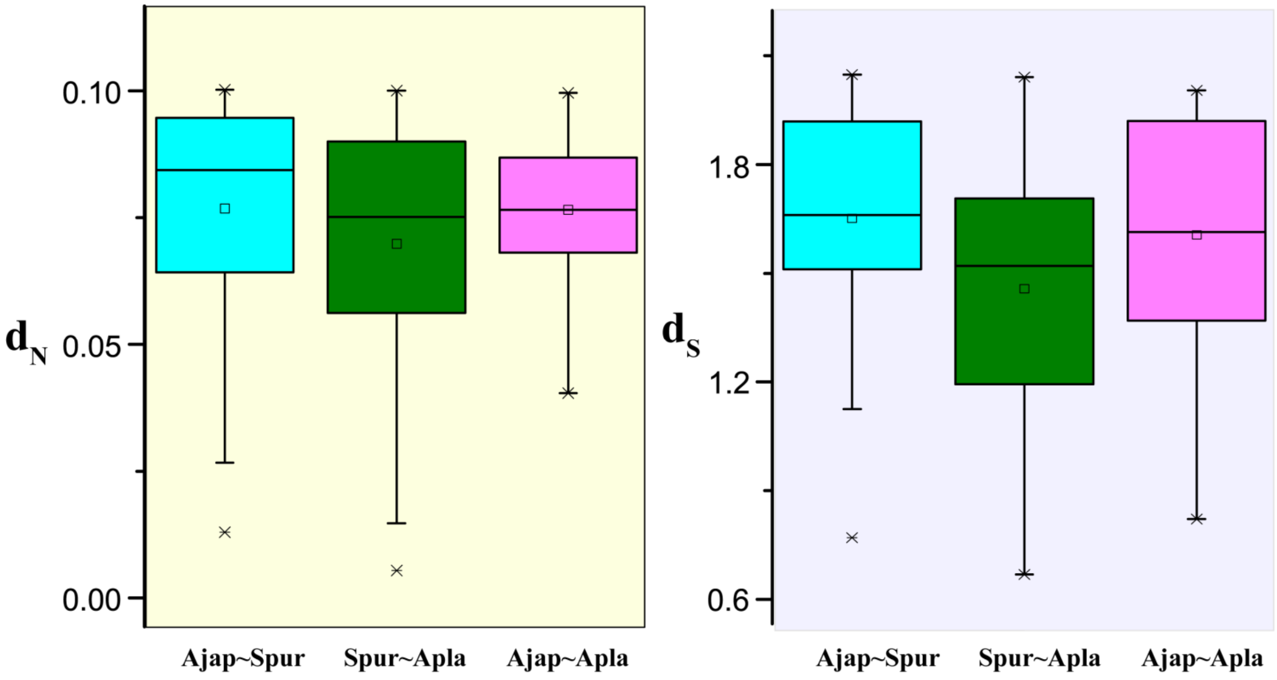

Supplementary Fig. S3. Frizzled gene family in echinoderms. (A) Phylogenetic tree of the Frizzled gene family of three echinoderms and *S. kowalevskii*. The phylogenetic tree is constructed using the maximum likelihood (ML) method with 1000 bootstraps and Bayesian inference (BI). Yellow circles indicate the support values of ML analysis larger than 80%. The support values of BI analysis are displayed beside each node. (B) Comparison of Frizzled gene family members across echinoderms and *S. kowalevskii*. The overlapping boxes represent duplicated Frizzled genes.

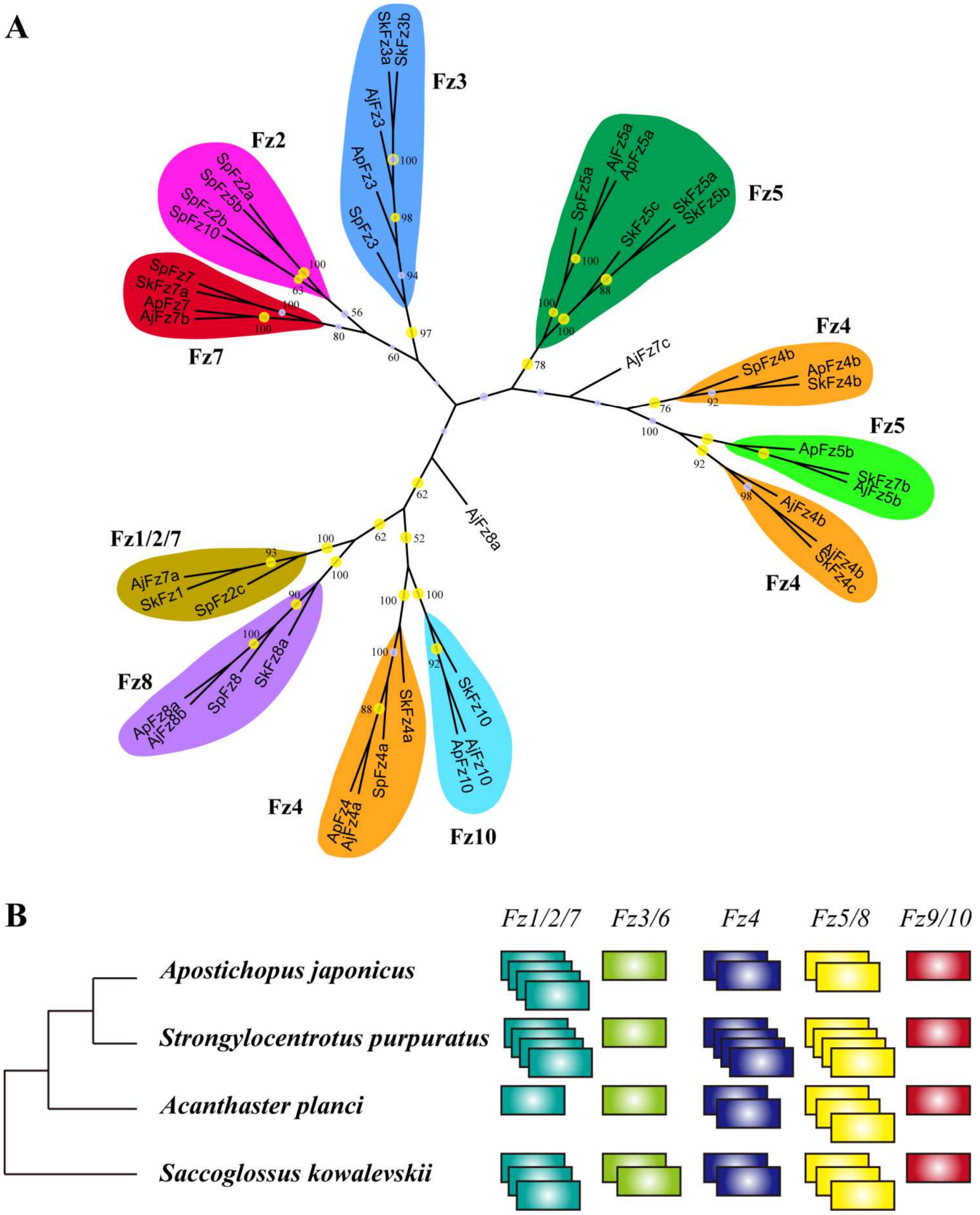

Supplementary Fig. S4. KEGG enrichment analysis of all upregulated genes during intestinal regeneration. Significantly enriched pathways of all DEGs. RichFactor is the ratio of the number of DEGs in this pathway term to the number of all genes in this pathway term.

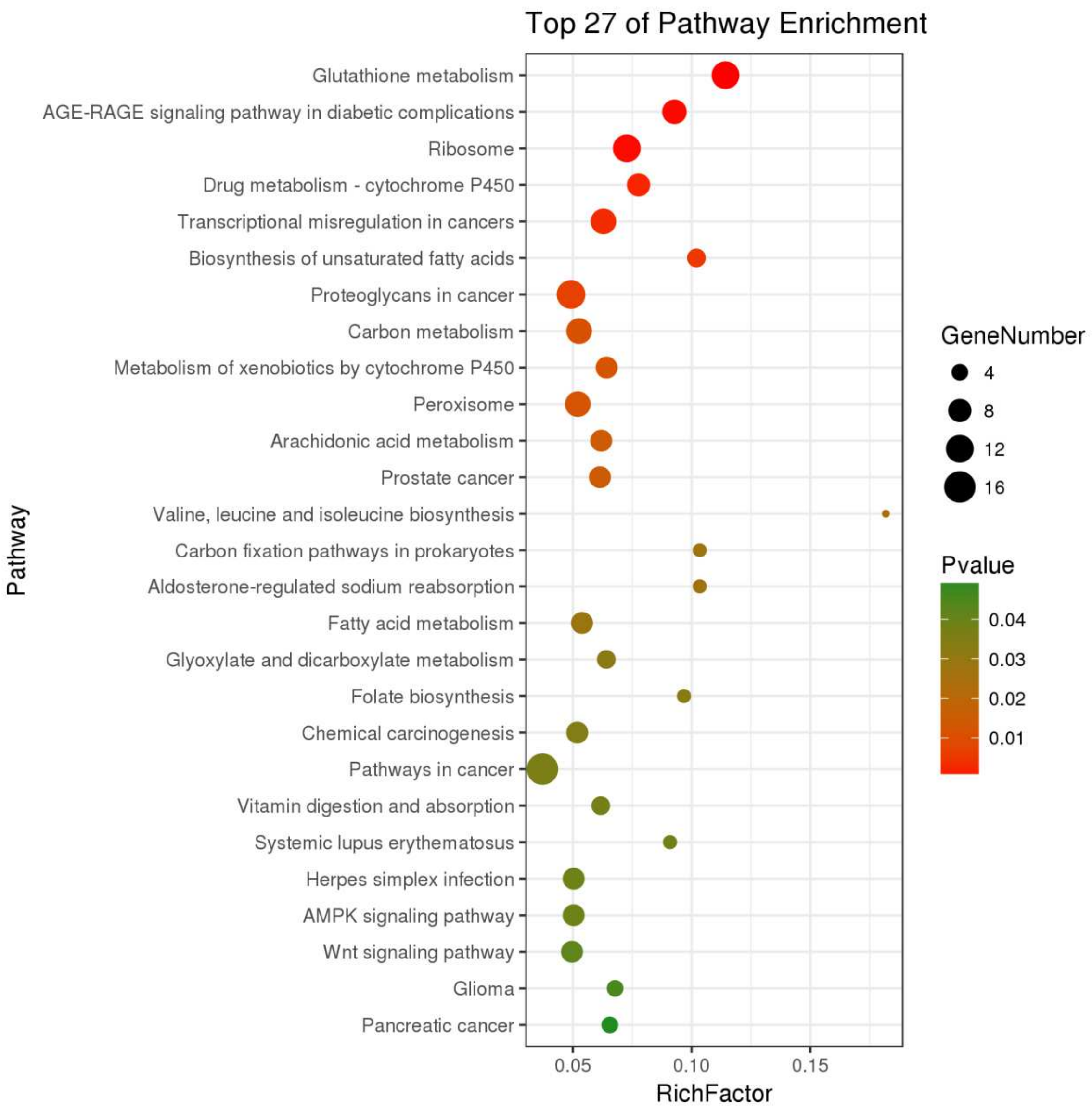

Supplementary Fig. S5. The expression pattern of the differentially expressed genes during intestinal regeneration. All the DEGs were divided into five groups, namely the DEGs that significantly upregulated at 3 day (3d), 5 day (5d), 7 day (7d), 14 day (14d), 21 day (21d), according to the Fig.4. The DEGs from the group of 3d were highly expressed during the whole regeneration stages, which was different from other groups.

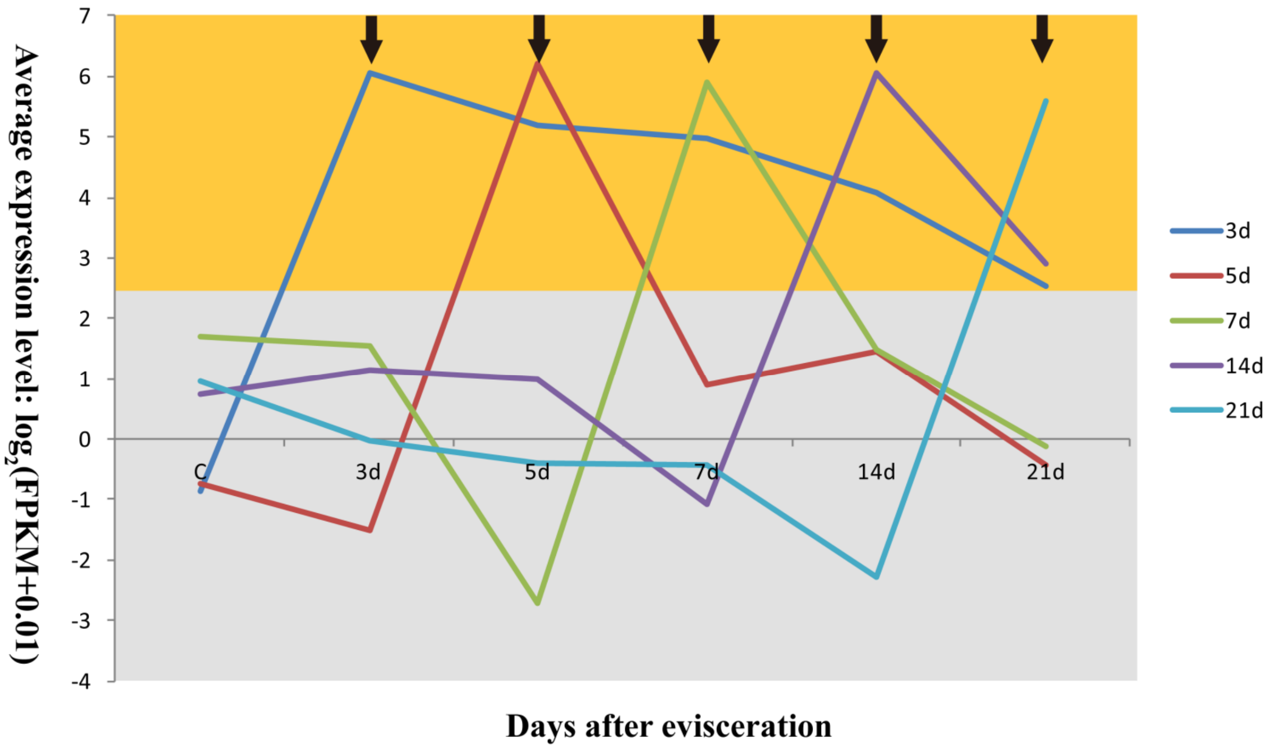

Supplementary Fig. S6. The expression patterns of some genes in the Wnt signaling pathway. The expression value are calculated as log_2(FPKM(i)/FPKM(C)), where FPKM(C) indicated the FPKM value of Control, and FPKM(i) indicated the FPKM value at different intestinal regeneration time points: 0, 3d, 5d, 7d, 14d, 21d.

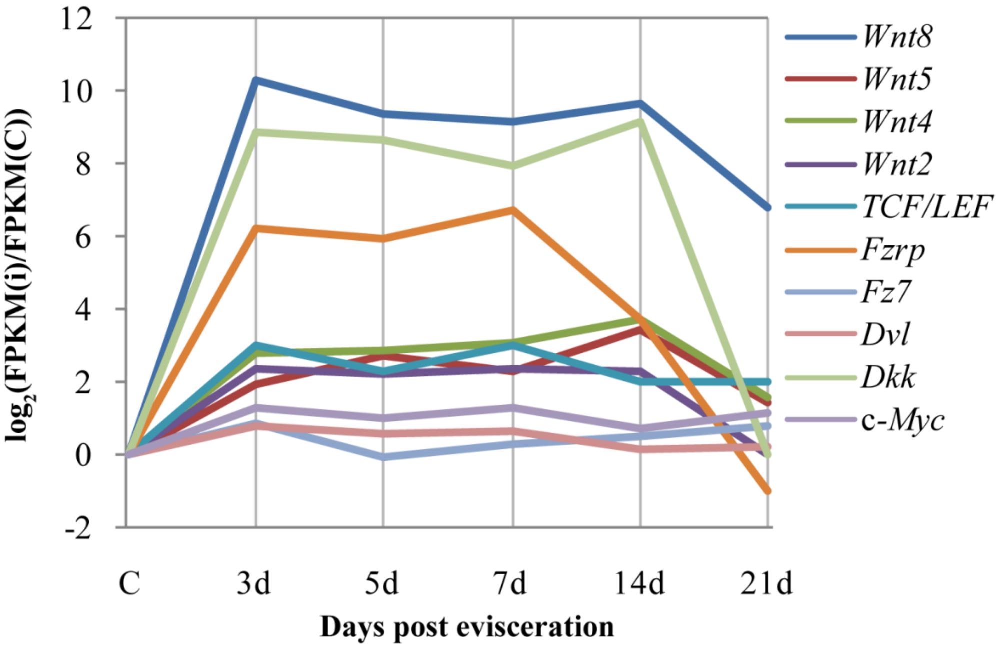

Supplementary Fig. S7. The results of silencing efficiency test. Relative expressions of three doses were detected at 48 hours after knockdown of the corresponding gene. ds*EGFP* injection was used as negative control treatments. The relative expression levels of *Wnt7* and *Dvl* were normalized against that of NADH. Data are the mean ± standard deviation of the triplicate experiments. Different lowercase letters indicate significant differences ($p < 0.05$).

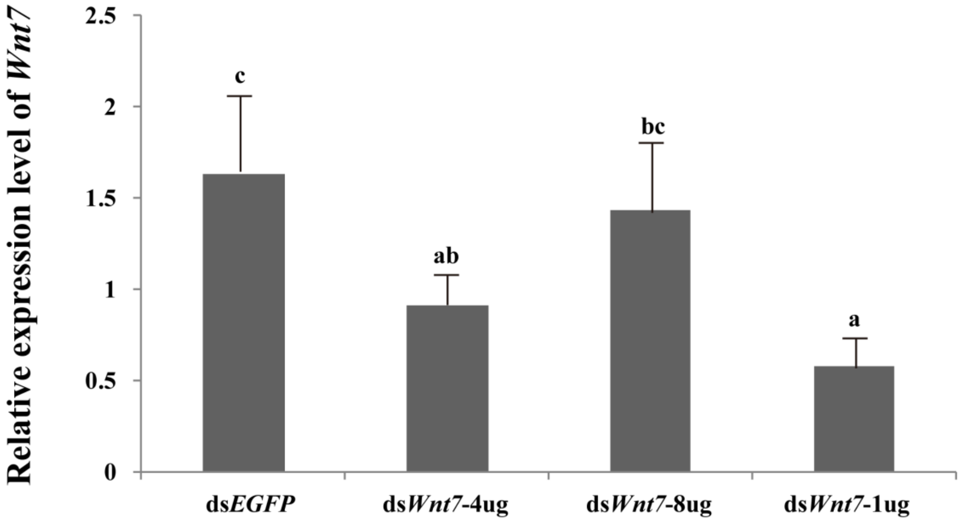

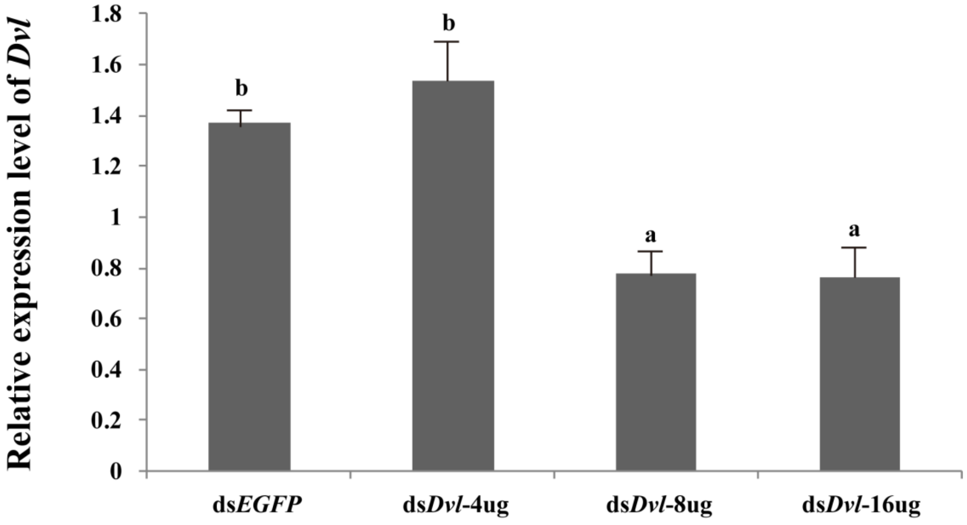

Supplement: Supplementary file 1 [file Presentation_1.PDF]
